# How Investors Attitudes Shape Stock Market Participation in the Presence of Financial Self-Efficacy

**DOI:** 10.3389/fpsyg.2020.553351

**Published:** 2020-10-22

**Authors:** Muhammad Asif Nadeem, Muhammad Ali Jibran Qamar, Mian Sajid Nazir, Israr Ahmad, Anton Timoshin, Khurram Shehzad

**Affiliations:** ^1^Department of Management Sciences, COMSATS University Islamabad (CUI), Lahore Campus, Lahore, Pakistan; ^2^School of Business Management, Universiti Utara Malaysia, Sintok, Malaysia; ^3^I.M. Sechenov First Moscow State Medical University (Sechenov University), Moscow, Russia; ^4^School of Economics and Management Science, Southeast University, Nanjing, China

**Keywords:** money attitudes, stock market participation, risk attitudes, financial self-efficacy, financial knowledge

## Abstract

The purpose of this study is to investigate how investor’s money attitudes shape their stock market participation (SMP) decisions. This study followed the theory of planned behavior (TPB), and a survey was conducted to collect the responses from active investors. Structural equation modeling (SEM) was used for the analysis of proposed relationships among the constructs, and a confirmatory factor analysis (CFA) was conducted to check the interrelation of the variables and validity of the constructs. This research has concluded that investor’s money attitudes are significant to affect their stock market participation decisions. Further, it was found that risk attitudes partially mediate the relationship between money attitudes and stock market participation. Moreover, financial knowledge and financial self-efficacy positively moderated the relationship between money attitudes and stock market participation. This research is one of the early attempts at studying the money attitudes of investors and introduces financial self-efficacy as a moderating construct between money attitudes and stock market participation. The sample size for this study was 250 respondents which can be increased in future research, and the same relationships can be tested by using a larger sample. Moreover, this study has used money attitudes as predictors of stock market participation. Still, many other variables, like personal value, can also be taken to investigate their influence on stock market participation.

## Introduction

Behavioral finance is knowing investor’s psychology related to financial decisions and is a combination of two disciplines, i.e., psychology and economics. This combination clarifies why and how people make irrational financial decisions when they save, invest, spent, and borrow money (Belsky and Gilovich, 1999). It is a blend of personal and social psychology principles with traditional finance theory to investigate and emphasize the stock market performance. Behavioral finance theory relies on how the thinking process and cognitive errors impact investor choice and prices of the stock exchange (Dam, 2017). Investors do not follow the rational models of investment which are assumed in the theory of efficient markets and there exist significant variations in the behavior of investors (Wärneryd, 2001; Riitsalu and Murakas, 2019).

For quite a long time, studies have been attempting to get a better understanding of stock market participation and the parameters impacting individual’s decisions whether or not participate in the stock market (Heaton and Lucas, 2000; Seasholes and Zhu, 2010; Korniotis and Kumar, 2011; Barber and Odean, 2013; Sivaramakrishnan et al., 2017; Bamforth et al., 2018; Ponchio et al., 2019). Previous studies have identified several factors that shape participation in the stock market, including demographics, education, social capital, income level, IQ level, investment knowledge, optimistic beliefs, financial literacy, peer effects, financial self-efficacy, stock market experiences, herding, heuristics, and cultural factors (Hong et al., 2004; Campbell, 2006; Brown et al., 2008; Georgarakos and Pasini, 2011; Grinblatt et al., 2011; Hurd et al., 2011; Malmendier and Nagel, 2011; Van Rooij et al., 2011; Bonaparte and Kumar, 2013; Calvet and Sodini, 2014; Kengatharan and Kengatharan, 2014; Li, 2014; Arrondel et al., 2015; Balloch et al., 2015; Gao, 2015; Gao et al., 2019; Liivamägi et al., 2019; Zou and Deng, 2019).

Individuals invest in the stock market to save their income for retirement (Clark-Murphy and Soutar, 2004). Stock market participation alludes to investing in the stock exchange by purchasing the shares of companies to increase wealth. Investments are committing money in an organization for a specific period with the aim of getting a return on it (Reilly and Brown, 2011). Stock market participation has extraordinary significance as it helps asset accumulation, welfare, and consumption smoothing (Cole and Shastry, 2009). Lack of stock market participation leads to welfare losses being imposed on the economy (Cocco et al., 2005). Different research has investigated the psychology of investing in the stock market (Wäneryd, 2001). Behavioral preferences and beliefs have been shown to significantly affect stock market participation (Dimmock and Kouwenberg, 2010; Georgarakos and Pasini, 2011). Demographics and background risk factors have a significant impact on stock market participation (Campbell, 2006).

Participation in financial markets has increased sharply recently (Van Rooij et al., 2011; Calvet et al., 2016). Exploring the causes of why individuals avoid stock market participation is crucial both on an individual and aggregate level (Luotonen, 2009) and it has become essential to investigate the factors that influence stock market participation. Literature has indicated that distinctive psychological factors impact stock market participation like investor’s beliefs, preferences, and psychological biases (Hilton, 2001; Daniel et al., 2002). A comprehensive set of traits clarifies the level of investments using stock market participation, although stock market literacy takes on a predominant role as indicated by Balloch et al. (2014). Literature has shown that variations in finance level and risk aversion do not agreeably explain investor’s choices whether to invest or not (Conlin et al., 2015).

As stock investment is generally talked about among individuals, the vast majority have built up specific attitudes about stock investment. Investment attitudes are fundamental for differentiating beginner investors who have not had investment experience yet, thus have not built any behaviors related to investment strategies. Attitudes anticipate behavior effectively when there is a high correspondence between the attitude object and the behavioral option (Tang and Baumeister, 1984; Grant and Beck, 2008). Adam and Shauki (2014) contended that under sensible considerations, individual’s attitudes significantly impact their sustainable investment decisions. Previous research related to financial problems revealed that money attitudes significantly change an individual’s financial management and the level of economic well-being (Shim et al., 2009; Phan et al., 2019). Likewise, money attitudes are viewed as critical when making investment decisions (Furnham, 1984; Wood and Zaichkowsky, 2004; Keller and Siegrist, 2006b). Choices taken in terms of money depend on money behavior which is the outcome of the effect of money attitudes. Individual money attitudes depend on various components, for instance, a person’s childhood experience, education, financial, and societal position. Based on these segments, money attitude differs from individual to individual.

Evidence recommends that the money behavior of investors should be developed based on these money attitudes (Roberts and Jones, 2001), as supported by the findings of Keller and Siegrist (2006b) and Dowling et al. (2009) that the investor’s financial decisions are based on money attitudes (Klontz et al., 2011). Previous studies defined monetary intelligence (MI) as individuals’ money attitudes to elaborate techniques to achieve financial happiness (Rose et al., 2016; Tang, 2016). Wood and Zaichkowsky (2004) investigated the attitude and trading behavior of investors and categorized them into four sections, i.e., risk intolerant, confident, less risk-averse young, and conservative long term investors.

Risk attitudes additionally explained stock market participation, for example, uncertainty dispersion, investors affinity to bet, the presence of a significant negative wealth shock, religion-incited betting attitudes, disclosure of corporate extortion in the society, and enormous hedging potential (Bonaparte et al., 2014; Giannetti and Wang, 2016). Individual’s risk attitudes are essential for deciding investment decisions (Barsky et al., 1997; Dimmock and Kouwenberg, 2010; Kumar et al., 2011; Giannetti and Wang, 2016). This research has considered risk attitudes to clarify the puzzle in stock market participation. This study intends to identify the influence of risk attitudes between the relationship of money attitudes and stock market participation focusing on the results of the research (Barsky et al., 1997) that an individual’s level of risk-taking in one place predicts risky behavior in another place.

Further, the literature has shown that cognitive ability essentially influences stock market participation, for instance, high financial literacy and a person’s intelligence quotient. Lapp (2010) inferred that a higher financial self-efficacy level leads to fewer financial problems. Fox and Bartholomae (2008) described financial self-efficacy as “knowledge and ability to affect and control one’s money related issues.” Besides, perceived behavioral control (which incorporates FSE) predicts positive monetary practices (Xiao et al., 2014). Likewise, Falahati and Paim (2011) considered financial knowledge as a critical component to improve behavior related to finance, thus influencing monetary prosperity (Saurabh and Nandan, 2018). Perry and Morris (2005) reasoned that financial knowledge emphatically impacts people’s economic behavior as financially literate people will exhibit more responsible financial behavior. Further, other individual attributes impacting stock market participation like age, gender, wealth, risk aversion, and education are also discussed in the literature (Georgarakos and Pasini, 2011; Almenberg and Dreber, 2015; Arts, 2018).

According to the best of the researcher’s knowledge, very little research has been found that focuses on investor’s money attitudes to explain their stock market participation (Keller and Siegrist, 2006a). Specifically, this research broadens the thought of monetary intelligence and investigates the degree to which investors adopt their money attitudes to “frame” (Tversky and Kahneman, 1981) effects of stock exchanges. The relationship between attitudes and behavior has been concentrated widely, yet research on money attitudes and stock market participation behavior is less abundant. This investigation can offer a new understanding that can be an essential expansion to the knowledge that previously exists. Thus, this research can fill the gap in research that explains how investor’s money attitudes affect stock market participation behavior (theory of planned behavior, i.e., TPB) and to overcome the difference between stock volatility and behavioral finance, and monetary intelligence. Money attitudes can be perceived in clarifying stock market participation and are an emerging research issue in behavioral finance. In this manner, it appears to be sensible to ask whether money attitudes may play a role in stock market participation.

## Literature Review

Ajzen (1985) proposed the theory of planned behavior (TPB) which was extracted from the theory of reason actions (Ajzen and Fishbein, 1980). The TPB lies among the valid models that explain human behavior (Ajzen, 1991). This theory states human behavior is affected through motivational factors like attitudes and perceived behavioral control. Attitudes can be expressed as “the degree to which an individual derives a positive or negative valuation from performing a specific behavior” (Ajzen, 1991). Further, the TPB intends to forecast conduct which is not entirely volitional by variable, for example, perceived behavioral control (Ajzen, 1991). This research follows the theory of planned behavior (TPB) which points out “*individual’s attitudes to behavior, subjective norms, and perceived behavioral control significantly affect their behavior intentions and behaviors*.”

Additionally, this theory primarily deals with attitude—a part of behavioral finance that assumes a critical job in stock market participation. A number of research has used the TPB to predict investor’s money attitudes in participating in financial markets and offered a basis to apply the TPB in stock market participation. The theory of planned behavior (TPB) focuses on the intentions of the individuals in performing specific behaviors. As indicated by this theory, determinants of intentions are attitudes, subjective norms, and perceived behavioral control. Attitudes show the level of an individual’s evaluation of behavior which can be favorable or unfavorable. Likewise, the subjective norm is perceived as social pressure in performing or not performing a specific behavior. Perceived behavioral control refers to control (ease or difficulty) of an individual in performing a specific behavior. Generally, favorable attitudes to specific behavior lead to a strong intention to perform that behavior (Ajzen, 1991; Ajzen and Driver, 1992).

### Money Attitudes and Stock Market Participation

Money attitudes can be defined as people’s attitudes that portray behavior in money matters (Klontz et al., 2011). People build up attitudes toward money on the premise of circumstances and experiences that they encounter over their lifetime. Money attitudes have four dimensions, i.e., money avoidance, money worship, money status, and money vigilance (Klontz et al., 2011). Individuals have different attitudes regarding money, for example, some people like it a lot (money worship), others do not take interest in money (money avoidance), a few people want to increase their status through money (money status), and others consider money as a source of shame (money vigilance). Money avoidance refers to believing that money is bad, that wealthy individuals are greedy and that they do not deserve money. Individuals may avoid spending money on even sensible or essential purchases. Individuals believing in the money worship dimension are convinced that more cash will solve the majority of their issues, that there will never be a sufficient amount, and that cash brings power and happiness. Individuals with money status dimensions see a clear distinction between socio-economic classes. Status lovers believe that owning the best and most current things gives status. In the money vigilance dimension, individuals consider that money is a profound source of shame and mystery, whether one has a lot or a little. The money vigilance element appears to be connected to alertness, readiness, watchfulness, and worry about money, and the feeling that one must be aware of pending inconvenience or threat (Klontz et al., 2011).

Literature has demonstrated that investors tempted by big returns have lost their money like in the 2008 Asia financial storm which shows that financial decisions are more complex when compared to past occurrences. Despite the fact that investors might acquire financial knowledge there still exists confusion about investing more appropriately. Along these lines, financial decisions require more accurate judgments on the part of investors. Recently, many studies have focused on psychological factors like cognitive abilities that affect stock market participation (Christelis et al., 2010). Likewise, attitudes toward money can be considered an important factor influencing stock market participation decisions as supported by Klontz et al. (2011) and Shih and Ke (2014). The literature on financial behaviors has focused more on exploratory and descriptive analyses and little attention has been given to aspects like attitudinal theoretical foundations.

Literature has shown that money attitudes have a significant influence on stock market participation and financial behaviors (Furnham, 1984; Chang and Hanna, 1992; Tang, 1992; Watson, 2003; Wood and Zaichkowsky, 2004; Canova et al., 2005; Perry and Morris, 2005; Keller and Siegrist, 2006b; Shim et al., 2009; Gambetti and Giusberti, 2012; Phan et al., 2019). Medina et al. (1996) have concluded that money is an important part of an individual’s life and it motivates the behaviors of people in different ways. Literature has affirmed that different investor groups having distinct money attitude types when investing in different financial assets (Wood and Zaichkowsky, 2004). The important money attitude scales discussed in the literature are the money attitudes scale (MAS), the money beliefs and behaviors scale (MBBS), and the money ethic behavior scale (MES) (Yamauchi and Templer, 1982; Furnham, 1984; Tang, 1992). Häusler et al. (2018) studied beliefs and stocks trading behavior and concluded that actions in the anterior insula while judging risky and safe decisions in investment activity are correlated with the stock trading behaviors of the individuals.

Likewise, money attitudes have a significant impact on an individual’s investment decisions (Furnham, 1984; Wood and Zaichkowsky, 2004; Keller and Siegrist, 2006b). Further, money attitudes also significantly influence individual’s financial management and economic well-being (Shim et al., 2009; Phan et al., 2019). Money attitudes shape investor’s money behavior (Roberts and Jones, 2001; Keller and Siegrist, 2006b; Dowling et al., 2009) while investor’s financial decisions are based on money attitudes (Klontz et al., 2011). Akhtar and Das (2019) have used the theory of planned behavior (TPB) and concluded that attitude partially moderates the relationship between financial knowledge and investment intentions.

People have turned out to be increasingly active in stock markets, and participation has been advanced by the introduction of new monetary products and services. However, a portion of these products is hard to grasp, particularly for monetarily unsophisticated investors. Standard models of portfolio choice consider that knowledgeable investors make rational decisions to augment lifetime utility. There are various motivations to presume that one’s choice about whether to put resources into stocks might be impacted by one’s money attitudes that are created through social interaction, education, and experience. The literature discussed has shown distinct insights into money attitudes, financial literacy, and financial behaviors; their applicability to Pakistan is limited. To date, little research has been conducted to investigate the influence of money attitudes of investors on their stock market participation decisions (Keller and Siegrist, 2006a). This study has a specific focus on attitudes toward money (money attitudes) of investors and the influence of these money attitudes on their stock market participation decisions. This research has developed a conceptual model that explains the psychological process of investor’s stock market participation.

H_1_: There is a significant impact of money attitudes on stock market participation of investors.

### Financial Knowledge and Stock Market Participation

Financial knowledge can be defined as “an individual’s knowledge and understanding of financial concepts” (Fox et al., 2005). Financial decision making is influenced by a person’s level of financial knowledge since people with a low level of financial literacy are less inclined to invest in stocks and consequently are less likely to take part in the stock exchange (Van Rooij et al., 2011). Further, it has been demonstrated that the probability of partaking in the financial exchange increases if a person is financially literate (Kaustia and Torstila, 2008). Financial knowledge is decisive for creating wealth (Van Rooij et al., 2012) consider this feasible via stock market participation. Bayer et al. (2009) have concluded that financial education leads to increased participation in the stock market. Bayer et al. (2009) has concluded that financial education leads to increased participation in the stock market. Literature has affirmed the moderating role of financial knowledge in financial behaviors including stock market participation (Morrin et al., 2012; Aren and Aydemir, 2015; Hayat and Anwar, 2016; Aydemir and Aren, 2017; Hadi, 2017; Shusha, 2017).

Some studies have preferred the moderating role of financial knowledge as compared to the direct effect on risky behaviors like investment decisions (Aydemir and Aren, 2017). Other studies have indicated that there are both negative and positive moderating roles of financial knowledge on the relationship between behavioral biases and investment decisions (Hayat and Anwar, 2016). Further, financial knowledge has moderated the relationship between emotional intelligence and investment decisions (Hadi, 2017). Aren and Aydemir (2015) have concluded that financial literacy has a moderating effect on the relationship between an individual’s factors and risky investment intentions. Moreover, literature has also confirmed that financial literacy moderates the relationship between demographic characteristics and financial risk tolerance (Shusha, 2017).

The researchers enhanced their argumentation by demonstrating that financially educated people face lower costs for gathering and handling information and consequently face a more moderate financial threshold for stock market participation. It has been investigated that both knowledge and attitudes may change behavior and knowledge may bring variation in attitudes and similarly it may also bring change in behavior via attitudes (Fessler et al., 2019) which indicates that for most people knowledge and attitudes may be considered as complementary rather than a substitute. Further, it has been shown that financial knowledge significantly impacts financial behavior and attitudes might have a significant role in shaping an individual’s financial behaviors. Moreover, individuals with greater knowledge scores possess higher attitudes scores (Fessler et al., 2019). Aydin and Akben Selcuk (2019) concluded that individuals with higher financial knowledge show favorable economic attitudes. Financially knowledgeable individuals exhibit responsible financial behavior (Fox et al., 2005) and individuals with low financial knowledge have a lower tendency to make risky investments such as in stocks (Van Rooij et al., 2011). Literature has indicated that many individuals lack knowledge about fundamental financial concepts (Lusardi and Mitchell, 2008). Due to limited knowledge regarding investments individuals are less likely to make informed financial decisions (Chen and Volpe, 1998).

Empirical investigations have demonstrated that education, financial knowledge, and risk tolerance firmly relate to stock market participation (Cole et al., 2012). Financial literacy increases the probability of participation in the stock market (Deng, 2019). It significantly benefits the investors in helping them to minimize the entry barriers to participate in derivative markets (Yu-Jen Hsiao, 2018). The literature has shown that attitudes and knowledge significantly influence consumer’s financial planning (Weisfeld-Spolter et al., 2018). Financial knowledge has a significant positive influence on financial attitude and behavior and attitude mediates the relationship between knowledge and behavior (Fessler et al., 2019). However, there is very little research in developing economies where these variables are linked with SMP (Sivaramakrishnan et al., 2017). Van Rooij et al. (2011) concluded that financial knowledge positively impacts stock market participation, and consequently, people with low financial knowledge are less likely to take an interest in the stock exchange. These findings cause concerns since people these days increasingly need to depend on themselves regarding significant financial decisions and the financial knowledge of young people is worryingly low (Lusardi et al., 2010). Based on the literature discussion on the role of financial knowledge, it seems sensible to analyze the moderating role of financial knowledge on the relationship between money attitudes and stock market participation.

H_2_: Financial knowledge moderates the relationship between money attitudes on stock market participation.

### Financial Self-Efficacy and Stock Market Participation

As indicated by the social cognitive theory of self-regulation, an individual’s higher self-efficacy level increases their probability of participation in a particular behavior, mostly positive monetary behavior, and makes them less inclined to feel money related pressure. Further, self-efficacy is the base of the activity of control and profoundly affects behavior (Bandura, 1991). Self-efficacy refers to an individual’s ability to control, manage, and impact different parts of his or her life. In this study, financial self-efficacy is characterized as a person’s perceived ability to control his/her finances. Individuals with more prominent self-efficacy over a specific conduct will generally participate in that conduct, plan higher objectives, show a constructive valuation of the job at hand, and show less dangerous pessimistic mental consequences (nervousness, stress, misery) related to adversity (Bandura, 1991, 1999). Self-efficacy must be assessed by the behavioral life domain that is under investigation (McAvay et al., 1996; Bandura, 1997).

Financial self-efficacy (FSE), a significant psychological construct, plays a significant role in shaping an individual’s decision-making style during different phases of life and personal finance behavior (Farrell et al., 2016; Asebedo and Payne, 2018). It differs from person to person (Dietz et al., 2003). Financial self-efficacy has shown both mediating and moderating roles in the relationship between personality traits and investment intension (Akhtar and Das, 2019). Literature has confirmed the moderating role of financial self-efficacy in financial behaviors including stock market participation (Lim et al., 2014; Qamar et al., 2016; Faison, 2019). FSE has a positive influence on risk-taking within investment portfolios (Montford and Goldsmith, 2016). Rothwell et al. (2016) have concluded that only financial knowledge is not enough for building financial capabilities, financial self-efficacy has also significant importance. In this manner, FSE may serve a significant role in stock market participation decisions.

Individuals with greater FSE better control and deal with their financial circumstances. When the market experiences volatility, investors with greater FSE typically hold their feeling of long-term control over their monetary circumstance than investors with low FSE. Literature has shown that FSE positively affects financial practices (Shim et al., 2012; Farrell et al., 2016). Based on the evidence on the role of financial self-efficacy, there is a need to investigate whether financial self-efficacy plays a moderating role in the relationship between money attitudes and stock market participation.

H_3_: Financial self-efficacy moderates the relationship between money attitudes on stock market participation.

### Risk Attitudes and Stock Market Participation

According to the traditional supposition, investors differ in their levels of risk aversion and different factors likewise influence their investment decisions (Grinblatt et al., 2011). Literature has affirmed that risk attitudes have a significant influence on stock market participation decisions (Wärneryd, 1996; Tigges et al., 2000; Clark-Murphy and Soutar, 2004; Wood and Zaichkowsky, 2004; Nosić and Weber, 2010; Wanyana et al., 2011; Zhang and Li, 2011; Barasinska et al., 2012; Noussair et al., 2013). It has also been investigated that less risk aversion predicts participation in various models (Haliassos and Bertaut, 1995). Further, risk attitudes mediate the relationship between social capital and stock market participation (Cheng et al., 2018). Saurabh and Nandan (2018) have confirmed the mediating role of risk attitudes toward financial satisfaction. Barsky et al. (1997) utilized responses from hypothetical questions to anticipate real-life risky behaviors, for example holding stocks and found little impact. Generally, willingness to taking risks leads to risky behaviors (Gürdal et al., 2017). Nosić and Weber (2010) concluded that a subjective risk attitude impacts investment in shares positively as supported by Cheng et al. (2018). Conversely, Sutter et al. (2013) found that risk attitudes weakly predict field behavior (Brown et al., 2008; Gürdal et al., 2017). Akhtar and Das (2019) studied the investment intentions of investors and concluded that high-risk individuals have greater investment intentions.

Some studies have indicated that individuals who take more risks invest in stock more often than those who take fewer risks (Clark-Murphy and Soutar, 2004). Similarly, the investors who are risk-seekers tend to invest in stocks rather than bonds and those investors who play safely increasingly invest in bonds as compared to stocks (Keller and Siegrist, 2006b). The literature has shown that the individual’s abilities to ensure against risks have a significant influence on their investment decisions (Heaton and Lucas, 2000; Cocco et al., 2005; Niu et al., 2020). Similarly, risk tolerance is strongly related to stock market participation (Cole et al., 2012). Similarly, happier individuals have positive attitudes toward risk and they might prefer stock market participation (Rao et al., 2016). The above literature discussions have shown mixed results that do not provide a clear understanding of the role of risk attitudes. Therefore, it seems sensible to investigate that risk attitudes may have a mediating effect on the relationship between money attitudes (MA) and stock market participation (SMP).

H_4_: Risk attitudes mediate the relationship between money attitudes on stock market participation.

## Research Methodology

The sample for this study consisted of active investors from Pakistan and the data were collected from the Pakistan Stock Exchange. This study adopted the sampling method proposed by Kline (Kline, 2011). The respondents were approached personally by the researcher, and 250 valid questionnaires were received. This study utilized a convenience sampling technique to select the respondents. The reason behind using a convenience sampling method was the availability of the investors due to the fact that online trading facility investors do not visit stock exchanges regularly and perform trading from their homes. During the data collection process, the researcher visited the broker’s offices in the stock exchange to get information on the investors and during investors’ availability, the questionnaires were handed out. Five measurement scales were used, which included money attitudes, risk attitudes, financial knowledge, financial self-efficacy, and stock market participation. Further investors were assured that their information would be kept anonymous and data were collected from volunteer investors. Structural equation modeling (SEM) and confirmatory factor analysis (CFA) was used to check the relationships between constructs and their reliability and validity.

Money attitude was a second-order construct and consisted of four sub-dimensions, i.e., money avoidance, money worship, money status, and money vigilance, containing 49 items in the questionnaire. Klontz’s money attitudes scale was used to measure the money attitudes of the investors (Klontz and Britt, 2012). Table 1 shows the measurement items for the money attitudes scale.

**TABLE 1:** Money attitude dimension items with factor loadings.

**Constructs**	**Measurement items**	**Factor loadings**
Money avoidance (MA)	I do not deserve a lot of money when others have less than me.	0.649
	Rich people are greedy.	0.721
	It is not okay to have more than you need.	0.567
	People get rich by taking advantage of others.	0.547
	I do not deserve the money.	0.533
	Good people should not care about money.	0.512
	It is hard to be rich and be a good person.	0.638
	Most rich people do not deserve their money.	0.607
	There is a virtue in living with less money.	0.562
	The less money you have, the better life is.	0.573
	Money corrupts people.	0.713
	Being rich means you no longer fit in with old friends and family.	0.637
	The rich take their money for granted.	0.559
	You cannot be rich and trust what people want from you.	0.504
	It is hard to accept financial gifts from others.	0.565
Money worship (MW)	Things would get better if I had more money.	0.721
	More money will make you happier.	0.801
	There will never be enough money.	0.601
	It is hard to be poor and happy.	0.609
	You can never have enough money.	0.680
	Money is power.	0.676
	I will never be able to afford the things I really want in life.	0.614
	The money would solve all my problems.	0.658
	If you have money, someone will try to take it away from you.	0.762
	You can’t trust people around money.	0.753
Money status (MS)	Most poor people do not deserve to have money.	0.555
	You can have love or money, but not both.	0.695
	I will not buy something unless it is new (e.g., car, house).	0.616
	Money is what gives life meaning.	0.654
	Your self-worth equals your net worth.	0.632
	If something is not considered the “best,” it is not worth buying.	0.804
	People are only as successful as the amount of money they earn.	0.713
	It is okay to keep secrets from your partner around money.	0.554
	As long as you live a good life you will always have enough money.	0.531
	Rich people have no reason to be unhappy.	0.696
	If you are good, your financial needs will be taken care of.	0.777
	If someone asked me how much I earned, I would probably tell them I earn more than I actually do.	0.694
Money vigilance (MV)	You should not tell others how much money you have or make.	0.615
	It is wrong to ask others how much money they have or make.	0.736
	Money should be saved not spent.	0.693
	It is important to save for a rainy day.	0.721
	People should work for their money and not be given financial handouts.	0.771
	If someone asked me how much I earned, I would probably tell them I earn less than I actually do.	0.575
	You should always look for the best deal before buying something, even if it takes more time.	0.822
	If you cannot pay cash for something, you should not buy it.	0.776
	It is not polite to talk about money.	0.595
	I would be a nervous wreck if I did not have money saved for an emergency.	0.766
	It is extravagant to spend money on oneself.	0.565
	I would be embarrassed to tell someone how much money I make.	0.749

The instrument for financial knowledge was adopted from research (Perry and Morris, 2005) and consisted of six items. The risk attitudes questionnaire was comprised of eight items (Zhang et al., 2019). Further, the Lown scale of financial self-efficacy was adopted to measure the financial self-efficacy of the investors (Lown, 2011), which contained six items. Finally, the stock market participation scale which had eight items included questions that were coded from 1 to 5 and were adopted from Luotonen (2009). Table 2 shows the measurement items for the scales of financial knowledge, financial self-efficacy, risk attitudes, and stock market participation.

**TABLE 2:** Construct, measurement items, and factor loadings.

**Constructs**	**Measurement items**	**Factor loadings**
Stock market participation (SMP)	Stock markets are unpredictable, that is why I would not invest in stocks.	0.828
	I would invest a larger sum of money in stocks.	0.772
	The uncertainty of whether the markets will rise or fall keeps me from buying stocks.	0.606
	When I hear the word “stocks,” the term “possible loss” comes to mind immediately.	0.743
	I am willing to take financial risks in order to substantially increase my assets.	0.721
	In money matters, I tend to be willing to take risks.	0.757
	How many types of stocks (e.g., agriculture, cement, textile sectors) do you own on average? (1) Less than 2 (2) 2–4 (3) 5–7 (4) 8–10 (5) More than 10	0.768
	How much is your total investment in stock market annually? (1) Less than 100,000 (2) 100,000–300,000 (3) 300,000–500,000 (4) 500,000–700,000 (5) More than 700,000	0.599
Risk attitudes (RA)	Taking risks makes life more fun.	0.688
	My friends would say that I am a risk taker.	0.701
	I enjoy taking risks in most aspects of my life.	0.720
	I would take a risk even if it meant I might get hurt.	0.876
	Taking risks is an important part of my life.	0.694
	I commonly make risky decisions.	0.681
	I am a believer of taking chances.	0.862
	I am attracted, rather than scared, by risk.	0.554
Financial self-efficacy (FSE)	It is hard to stick to my spending plan when unexpected expenses arise.	0.771
	It is challenging to make progress toward my financial goals.	0.850
	When unexpected expenses occur, I usually have to use credit.	0.669
	When faced with a financial challenge, I have a hard time figuring out a solution.	0.706
	I lack confidence in my ability to manage my finances.	0.588
	I worry about running out of money in retirement.	0.760
Financial knowledge (FK)	Interest rates, finance charges and credit terms.	0.810
	Managing finances	0.815
	Investing money	0.624
	Debt card, credit card, cheque book, taxes.	0.622
	Common stocks, preferred shares, bonds, govt. securities	0.525
	Stock exchanges, mutual funds, insurance companies, microfinance institutions.	0.777

The money attitudes and risk attitudes were measured on a five-point Likert scale marked from “Strongly Disagree” to “Strongly Agree.” The questionnaire related to financial knowledge was measured on a five-point Likert scale marked from “Nothing” to “A Lot.” Similarly, the financial self-efficacy scale was measured on a five-point Likert scale marked from “Exactly True” to “Not at all True.” Table 3 shows the constructs with their items and references.

**TABLE 3:** Variables and scales.

**Variables**	**No. of items**	**References**
Financial knowledge	6	Perry and Morris, 2005
Money attitudes	49	Klontz et al., 2011
Financial self-efficacy	6	Lown, 2011
Risk attitudes	8	Zhang et al., 2019
Stock market participation	8	Luotonen, 2009

**FIGURE 1:**
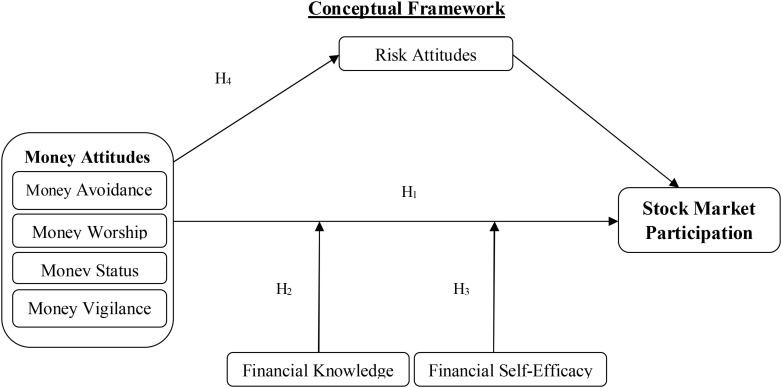
Conceptual framework.

This research investigated the influence of money attitudes on stock market participation by following money attitudes and financial practices research (Klontz et al., 2011). This literature review has indicated that variables risk attitudes, financial knowledge, and financial self-efficacy have a robust link with these variables, i.e., money attitudes and stock market participation. Therefore, this study intended to check the moderating role of financial knowledge and financial self-efficacy and the mediating effect of risk attitudes on the relationship between money attitudes and stock market participation. Demographic variables cannot be ignored in this research. Literature has confirmed that socio-demographic variables significantly influence financial behaviors (Furnham, 1984; Tang and Gilbert, 1995; Korniotis and Kumar, 2011). It has been shown that women participate less in the stock market when compared to men (Van Rooij et al., 2011). Similarly, household income positively influences household saving increments (Rha et al., 2006). Figure 1 shows the conceptual framework based on the objectives of this study and the literature review.

Descriptive statistics have explained how the data are distributed. Structural equation modeling (SEM) was run to check the influence of the predictor variable (money attitudes) on the outcome variable (stock market participation) and moderation and mediation effects. Confirmatory factor analysis was used to check the validity of the scales. The missing values and outliers were considered, and interestingly there were no missing values in the datasheet. For this study, the data were collected at the same time, which can cause common-method bias (Chang et al., 2010). Exploratory factor analysis (EFA) was performed to check the significant variance explained from the single factor to avoid common-method bias (Podsakoff and Organ, 1986). According to the result of EFA, it was found that one single factor was showing a 19.495% variance. Hence, common-method bias was not an issue. Table 4 shows the variables and their definitions.

**TABLE 4:** Definitions of variables.

**Sr. #**	**Variable name**	**Variable definition**
1.	Money attitudes	People’s attitudes which portray behavior in money matters. People build up an attitude toward money on the premise of circumstances and experiences that one encounters over a lifetime.
2.	Money avoidance	Believing that money is bad, that wealthy individuals are greedy, and that they don’t deserve money. Individuals may avoid spending money on even sensible or essential purchases.
3.	Money worship	Individuals with this characteristic are convinced that more cash will solve the majority of their issues, that there will never be a sufficient amount, and that cash brings power and happiness.
4.	Money status	People who trust that money is status see a clear distinction between socio-economic classes. Status lovers believe that owning the best and most current things gives status.
5.	Money vigilance	For some individuals, money is a profound source of shame and mystery, whether one has a lot or a little. The money vigilance element appears to be connected to alertness, readiness, watchfulness, and worry about money, and the feeling that one must be aware of pending inconvenience or threat.
6.	Financial self-efficacy	Financial self-efficacy is characterized as a man’s perceived ability to control his/her finance.
7.	Financial knowledge	Financial knowledge is understanding critical financial terms and ideas needed to function day by day in society.
8.	Risk attitudes	Risk attitudes are an individual’s attitudes toward risk-taking and consist of two types, i.e., risk aversion and risk-seeker. Risk aversion is the behavior of humans who, when exposed to uncertainty, attempt to lower that uncertainty. A risk-seeker or risk-lover is a person who prefers risk.

The convergent and discriminant validity were validated by checking the acceptable range of the AVE (average variance extracted). The value of the AVE (average variance extracted) should be greater than 0.5 to achieve the convergent validity and further, to reach the discriminant validity, the square root of the AVE was taken and placed in the diagonal to compare with the correlations of the variables. The diagonal values were more significant than the association, and in this way, discriminant validity was validated. Two measures were used, i.e., Cronbach’s Alpha and construct reliability (CR) to find the reliability of the scale. To confirm the reliability of the questionnaire, the value of Cronbach’s Alpha should be higher than 0.7, and the value of CR (construct reliability) should be greater than 0.6. The normality of the constructs was analyzed by using the P-P plots, which showed that the data were statistically normal. Therefore, the data were valid for the analysis purpose and met the assumption of normality.

## Results and Discussion

### Sample Profile

The demographics presented in Table 5 showed that the majority of investors who participated in this study were male, i.e., 241 (96.4%) male and 9 (3.6%) female investors. The number of investors who participated was 250 in which 64 (25.6%) were in the age range 20–30, 86 (34.4%) were between the age range 31–40, 73 (29.2%) were in the age range 41–50, 17 were aged between 51 and 60, and 10 investors were at an age greater than 60.

**TABLE 5:** Descriptive statistics of the variables.

**Variables**	**Definitions and frequency**		**Percent**	**Mean**	**SD**
Gender	1 = Male	241	96.4	0.96	0.187
	0 = Female	9	3.6		
Age	1 = 20–30	64	25.6		
	2 = 31–40	86	34.4	2.29	1.048
	3 = 41–50	73	29.2		
	4 = 51–60	17	6.8		
	5 = 60	10	4.0		
	Total	250			
Residential area	1 = Urban	220	88.0		
	2 = Rural	30	12.0	1.12	0.326
	Total	250			
Education	1 = Matric	3	1.2		
	2 = Intermediate	41	16.4	3.30	0.804
	3 = Bachelor	87	34.8		
	4 = Master	115	46.0		
	5 = MS	4	1.6		
	Total	250			
Monthly income	1 = 40,000	106	42.4		
	2 = 41,000–80,000	103	41.2	1.88	1.060
	3 = 81,000–120,000	19	7.6		
	4 = 121,000–160,000	8	3.2		
	5 = 160,000	14	5.6		
	Total	25			

*Source: Authors calculations.*

The majority of investors who participated in this research were between the ages of 31–40. Most of the investors were from urban backgrounds (220, 88.0%), while 30 (12.0%) investors were from rural backgrounds. Further, 115 (46.0%) investors had a master’s degree, while only three investors (1.2%) were educated to a Matric level. Forty-one investors (16.4%) had an intermediate education, 87 (34.8%) had a Bachelor’s degree, and four investors (1.6%) had an education level greater than MS. The monthly income of 106 (42.4%) of the investors was less than Rs. 40,000, 103 (41.2%) investors had a monthly income of between 41,000 and 80,000, 19 (7.6%) were between 81,000 and 120,000, 8 (3.2%) were between 121,000 and 160,000, and 14 (5.6%) investors had a monthly income greater than 160,000.

### Statistical Analysis

The statistical analysis of research models was performed through SPSS 22 and AMOS 26. Structural equation modeling (SEM) was applied using AMOS 26, and the effect of predictor, moderator, and mediator on the dependent variable was analyzed. Structural equation modeling (SEM) is useful when there are higher numbers of variables in the model, and it includes latent variables into the study and also calculates the measurement error (Hair et al., 2011).

### Confirmatory Factor Analysis (CFA), Reliability, and Validity

Confirmatory factor analysis (CFA) was applied to conform to the standards of convergent validity and discriminant validity of the constructs. Further, for testing the goodness of fit statistics model fit indices were chosen. The initial results of CFA showed the goodness of fit index (GFI) value as 0.967, which was higher than the required value, i.e., 0.90. The adjusted goodness of fit index (AGFI) value was 0.924, for the Tucker-Lewis index (TLI), the value was 0.964, for the comparative fit index (CFI) the value was 0.981, for the incremental fit index (IFI) the value was 0.981. The values of all these fitness indices was higher than 0.90, which is the required level. The value of RMSEA (root mean square error approximation) was 0.060, which was also in an acceptable range. Further, the value of R^2^ was 0.43, which showed that the predictor variable (money attitudes) brought a 43% variance in the outcome variable (stock market participation). The convergent and discriminant validity were validated by checking the acceptable range of the AVE (average variance extracted). Table 6 shows the results of CFA and reliability analysis.

**TABLE 6:** Confirmatory factor analysis and reliability.

**Constructs**	**Items with standard factor loadings**		**KMO**	**CR**	**Cronbach’s alpha**	**AVE**
Money avoidance (MA)	MA1 = 0.649 MA2 = 0.721 MA3 = 0.567 MA4 = 0.547 MA5 = 0.533 MA6 = 0.512 MA7 = 0.638 MA8 = 0.607	MA9 = 0.562 MA10 = 0.573 MA11 = 0.713 MA12 = 0.637 MA13 = 0.559 MA14 = 0.504 MA15 = 0.565	0.855	0.89	0.866	0.59
Money worship (MW)	MW1 = 0.721 MW2 = 0.801 MW3 = 0.601 MW4 = 0.609 MW5 = 0.680	MW6 = 0.676 MW7 = 0.614 MW8 = 0.658 MW9 = 0.762 MW10 = 0.753	0.777	0.90	0.768	0.69
Money status (MS)	MS1 = 0.555 MS2 = 0.695 MS3 = 0.616 MS4 = 0.654 MS5 = 0.632 MS6 = 0.804	MS7 = 0.713 MS8 = 0.554 MS9 = 0.531 MS10 = 0.696 MS11 = 0.777 MS12 = 0.694	0.822	0.90	0.825	0.66
Money vigilance (MV)	MV1 = 0.615 MV2 = 0.736 MV3 = 0.693 MV4 = 0.721 MV5 = 0.771 MV6 = 0.575	MV7 = 0.822 MV8 = 0.776 MV9 = 0.595 MV10 = 0.766 MV11 = 0.565 MV12 = 0.749	0.729	0.92	0.780	0.70
Stock market participation (SMP)	SMP1 = 0.828 SMP2 = 0.772 SMP3 = 0.606 SMP4 = 0.743	SMP5 = 0.721 SMP6 = 0.757 SMP7 = 0.768 SMP8 = 0.599	0.774	0.90	0.742	0.72
Risk attitudes (RA)	RA1 = 0.688 RA2 = 0.701 RA3 = 0.720 RA4 = 0.876	RA5 = 0.694 RA6 = 0.681 RA7 = 0.862 RA8 = 0.554	0.801	0.89	0.845	0.72
Financial self-efficacy (FSE)	FSE1 = 0.771 FSE2 = 0.850 FSE3 = 0.669	FSE4 = 0.706 FSE5 = 0.588 FSE6 = 0.760	0.651	0.87	0.596	0.72
Financial knowledge (FK)	FK1 = 0.810 FK2 = 0.815 FK3 = 0.624	FK4 = 0.622 FK5 = 0.525 FK6 = 0.777	0.732	0.85	0.664	0.70

*Source: Authors calculations. KMO, Kaiser-Meyer-Olkin; AVE, average variance extracted.*

To validate the convergent validity, the value of the AVE should be greater than 0.5 (Hair et al., 1998). For achieving the discriminant validity, the square root of the AVE was taken and placed in the diagonal to compare with the Pearson correlations of the variables and the correlations were less than 0.80 (Brown, 2015). Therefore, all the variables were fine for convergent and discriminant validity. Cronbach’s Alpha and construct reliability were used to find the reliability of the scale. The value of Cronbach’s Alpha should be greater than 0.7 for a reliable dataset (Hair et al., 1998). Construct reliability was measured through composite reliability which should be greater than 0.6 (Bagozzi et al., 1998). For discriminant validity, the square root of the AVE should be higher than the correlations of each construct (Chin et al., 1997). Therefore, Table 7 validates discriminant validity.

**TABLE 7:** Discriminant validity and correlations.

**Constructs**	**Money avoidance**	**Money worship**	**Money status**	**Money vigilance**	**Stock market participation**	**Risk attitudes**	**Financial self-efficacy**	**Financial knowledge**
Money avoidance	**0.74**							
Money worship	0.67**	**0.83**						
Money status	0.66**	0.67**	**0.81**					
Money vigilance	0.54**	0.56**	0.64**	**0.84**				
Stock market participation	0.50**	0.40**	0.55**	0.47**	**0.85**			
Risk attitudes	0.42**	0.30**	0.50**	0.47**	0.36**	**0.85**		
Financial self-efficacy	0.33**	0.35**	0.38**	0.31**	0.41**	0.36**	**0.85**	
Financial knowledge	0.39**	0.40**	0.31**	0.45**	0.52**	0.43**	0.39**	**0.83**
Mean	3.23	3.38	3.23	3.49	3.35	3.50	4.26	4.17
SD	0.68	0.65	0.64	0.52	0.83	0.67	0.65	0.85

Source: Authors calculations. The values in the diagonal are the square root of the AVE, and the off-diagonal values are the correlations among variables. The discriminant validity is achieved when the diagonal values are higher than the off-diagonal values. **Correlation is significant at 0.01 level (2-tailed). Bold values are the values in the diagonal are the square root of the AVE.

### Structural Model; Goodness of Fit Statistics

**FIGURE 2:**
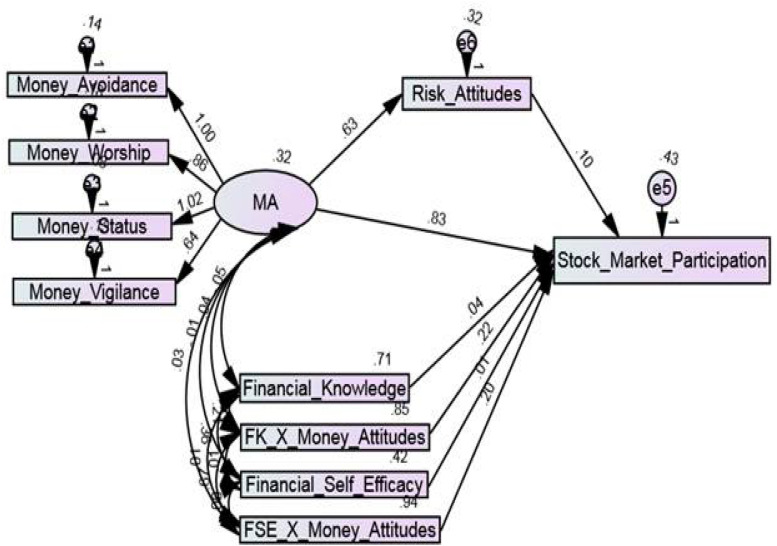
Structural model.

After ensuring the validity and reliability of the variables, the established relationships in the conceptual framework were tested (Figure 1). In the conceptual framework, the construct of money attitudes was a second-order construct and consisted of four dimensions, i.e., money avoidance, money worship, money status, and money vigilance. First of all, through these four dimensions, the second-order construct money attitudes was measured, and then the impact of money attitudes on stock market participation was analyzed. The modification indices were also analyzed for improving model fitness (Anderson and Gerbing, 1988). Most commonly used fit indices were used for the goodness of fit statistics (Table 8). The structural equation modeling (SEM) results showed that the structural model was fit, and that money attitudes brought a 43% variance in stock market participation of the investors (adjusted R^2^ = 0.43, i.e., 43%) (Table 8). Figure 2 shows the structural model using AMOS 26.

**TABLE 8:** Model fit statistics.

**Goodness of fit indices**	**Structural model (conceptual framework)**	**Norms**	**References**
X2	45.715	NA	
X2/df	1.905	>1 and <5	
GFI	0.967	≥0.90	Shevlin and Miles, 1998
AGFI	0.924	≥0.90	
TLI	0.964	≥0.90	Hu and Bentler, 1999
CFI	0.981	≥0.90	Hu and Bentler, 1999
RMSEA	0.060	≥0.05	MacCallum et al., 1996
IFI	0.981	≥0.90	Bagozzi and Yi, 1988
R^2^ Adjusted (SMP)	0.43		

*Source: Authors calculations.*

The results of the first hypothesis showed that money attitudes (MA) (β = 0.833, þ = 0.000) have a strong significant positive impact on the stock market participation (SMP) of the investors, and hence hypothesis H1 was supported. Therefore, this research shows the significance of money attitudes of investors in predicting stock market participation which means that the investors are more concerned about their money attitudes while deciding whether to participate in the stock market or not.

### Moderation Analysis

The moderation was tested in AMOS 26 by constructing a structured diagram. First of all, the independent variable money attitude and two moderating variables, i.e., financial knowledge and financial self-efficacy were standardized, and after these two interaction terms were computed using SPSS 22. The first interaction term (FK_X_Money_Attitudes) was calculated by multiplying the z-score of financial knowledge (FK) and money attitudes (MA). After computing the interaction term, it was entered into the model with the independent and dependent variables (Figure 2). The moderation model having a standardized effect of the interaction term, i.e., FK_X_Money_Attitudes (β = 0.219, þ = 0.007) with stock market participation, showed that financial knowledge positively moderated the relationship between money attitudes and stock market participation. It shows that higher financial knowledge strengthens this relationship, and more financially literate investors have greater stock market participation.

The second interaction term (FSE_X_Money_Attitudes) was computed by multiplying the z-score of financial self-efficacy and money attitudes. Further, the standardized effect of the interaction term, i.e., FSE_X_Money_Attitudes (β = 0.198, þ = 0.006) with stock market participation showed that financial self-efficacy also positively moderates the relationship between money attitudes and stock market participation. This finding has indicated that individuals with substantial control over their financial abilities are more likely to invest in the stock market. Therefore, financial knowledge and financial self-efficacy both strengthen the positive relationship between money attitudes and stock market participation in investors (Figure 2). Consequently, the hypotheses H_2_ and H_3_ were supported.

### Mediation Analysis

For testing the mediating role of risk attitudes, two approaches were used, i.e., Baron and Kenny (1986) and Hoyle and Smith (1994). First of all, the direct path between money attitudes and stock market participation was drawn, and the relationship between the predictor and the dependent variable was tested. After this, an indirect path was drawn as money attitudes-risk attitudes-stock market participation, and the mediating role of risk attitudes was analyzed. The direct path from money attitudes to stock market participation showed that money attitudes exerted a positive impact on stock market participation (β = 0.833, þ = 0.000). When the risk attitude was taken as a mediating variable, it showed partial mediation between the relationship of money attitudes and stock market participation (β = 0.096, þ = 0.046). Thus, risk attitudes partially mediate the relationship between money attitudes and stock market participation. This finding indicates that the individuals who are high risk-takers have a higher probability of participating in the stock market as supported by Akhtar and Das (2019). Hence hypothesis H_4_ was also supported.

## Discussion and Implications

As with many types of research carried out internationally, this study intended to identify the influence of investor’s money attitudes in their stock market participation decisions by collecting primary data to test the hypotheses. Based on data collected from investors and through structural equation modeling (SEM), this study has found that money attitudes were significant in predicting stock market participation of the individual investors (β = 0.833) supported by Furnham (1984); Wood and Zaichkowsky (2004), Keller and Siegrist (2006a), and Klontz et al. (2011). Support has been found from the theory of planned behavior (TPB) that individual’s attitudes have a strong influence on their behaviors (Ajzen, 1985). The results indicate that investors consider their attitudes toward money very important while participating in the stock market as supported by O’Connor and White (2010) and Schmidt (2010). Investor groups having distinct money attitude types that invest in different financial assets (Wood and Zaichkowsky, 2004). Further, findings have shown that women participate less in the stock market as compared to men as supported by Van Rooij et al. (2011). Following past literature, financial knowledge has been found to have a significant influence on stock market participation decisions of investors (Van Rooij et al., 2011) and positively moderate the relationship between money attitudes and stock market participation (β = 0.219) as supported by Aren and Aydemir (2015); Hadi (2017), and Shusha (2017). This shows that more financially literate investors have greater stock market participation as supported by Parrotta and Johnson (1998) and Perry and Morris (2005). The reason could be that when the investors have sufficient financial knowledge related to the stock market, industries, share, and bonds they are capable of making sound financial decisions, and similarly their stock market participation increases. Previous studies have shown both negative and positive moderating roles in financial knowledge (Hayat and Anwar, 2016) and some studies have preferred the moderating role of financial knowledge as compared to direct effects (Aydemir and Aren, 2017). This research has supported the findings of previous studies which have shown that financially knowledgeable individuals show responsible financial behavior and individuals with low financial knowledge have a lower tendency to make risky investments such as in stocks (Fox et al., 2005; Van Rooij et al., 2011).

The results have shown that financial self-efficacy moderates the relationship between money attitudes and stock market participation as supported by Lim et al. (2014); Faison (2019), and Wang (2019). It indicates that the investors who have greater control over their finances substantially participate in stock market activities as they trust in their financial capabilities (Wang, 2019). This shows that when the market experiences volatility, investors with greater FSE will typically hold their feeling of long-term control over their monetary circumstance than investors with low FSE. Therefore, financial self-efficacy, a significant psychological construct, can play a significant role in shaping an individual’s decision-making style during different phases of life especially in personal finance behaviors as supported by Farrell et al. (2016) and Asebedo and Payne (2018). Further, risk attitudes have partially mediated the relationship between money attitudes and stock market participation as supported by Cheng et al. (2018) and Saurabh and Nandan (2018). These results support the previous studies findings (Wood and Zaichkowsky, 2004) and indicate that the investors who are risk-seekers increasingly participate in the stock market as supported by Cheng et al. (2018) and Akhtar and Das (2019). It indicates that the investors who are risk-seekers tend to invest in stocks rather than bonds and those investors who play it safe increasingly invest in bonds as compared to stocks as supported by Keller and Siegrist (2006b). Further, an individual’s ability to ensure against risks significantly influences investment decisions (Cocco et al., 2005; Niu et al., 2020).

### Practical Implications

This research will help financial professionals, economic institutions, and policy makers to make better strategies and make financial decisions related to the stock market. This study has shown the importance of money attitudes of investors in their financial decisions related to stock market participation. Further, the important role of the variables financial knowledge, financial self-efficacy, and risk attitudes have been identified in this relationship. This study can benefit governments and stock market professionals who need to know about the important influence of money attitudes of investors in their investment decisions. More focus could be given to those factors that shape these money attitudes. It can help to better understand stock market participation and the parameters impacting an individual’s decisions whether or not to participate in the stock market. This study can also help in understanding that investment attitudes are essential for differentiating beginner investors who have not had investment experience yet, thus have not built behavior related to investment strategies. Further, this study has briefly explained the importance of variables like financial knowledge, financial self-efficacy, and risk attitudes which can also be focused by financial professionals in analyzing the investment behavior of the investors.

### Theoretical Implications

This study will add to the existing knowledge on the attitude and behavior relationship, as in previous studies the attitudes have been studied in a broader perspective and there is little research on the subtypes of attitudes like money attitudes and risk attitudes, especially in relation with stock market participation. This study has shown the importance of the money attitudes of investors and also their strong influence in their stock market participation decisions. A comprehensive set of traits clarifies the level of investments using stock market participation; money attitudes can take a predominant role. Further, attitudes anticipate behavior effectively just when there is a high correspondence between the attitude object and the behavioral option. Moreover, this study will add to the literature on the moderating role of financial knowledge and financial self-efficacy and the mediating role of risk attitudes on the relationship between money attitudes and stock market participation. This study has used the theory of planned behavior to investigate the attitude and behavior relationship, which has provided proof of the validity of the TPB. Specifically, this research broadens the thought of monetary intelligence through its investigation of the degree to which investors adopt their money attitudes to “frame” the effects of the stock exchange.

## Conclusion and Future Research Directions

This research is an attempt to better understand why and when investors decide to participate in the stock market and whether their participation decisions are differentiated by their risk attitudes, financial knowledge, and financial self-efficacy. This study has provided evidence that investor’s stock market participation decisions are influenced by distinct psychological factors like money attitudes, risk attitudes, and financial self-efficacy. This research is of great interest because it intends to describe not only the importance of money attitudes in stock market participation decisions but also to clarify the influence of other variables that mostly go unnoticed. From one perspective, the study fills the research gap present in previous studies that have not highlighted the psychological aspect of money attitudes for participation in the stock market. Further, this research explains the vital influence of intangible assets, for instance, risk attitudes and financial self-efficacy and resources, for example, financial knowledge importance for participating in the stock market.

Although this research contributes to existing knowledge, it has some limitations. Firstly, the sample size for this research was limited to 250 active investors, and the reason for this sample size was the availability of online access for trading. Due to online trading access, the investors are less likely to visit the stock market as they can trade from their respective locations. Therefore the sample size can be increased for a more in-depth understanding of these relationships in future studies. Secondly, this study has specifically focused on money attitudes as compared to previous studies which broadly studied attitudes, indicating the research gap. Hence, other subtypes of attitudes can also be considered in future studies. Other suggestions include finding the influence of socio-demographics in this relationship, comparative study explaining the differences among attitudes in different countries, and taking other moderating and mediating variables to enhance the predictive power of the model.

## Data Availability Statement

The raw data supporting the conclusions of this article will be made available by the authors, without undue reservation, to any qualified researcher.

## Ethics Statement

The studies involving human participants were reviewed and approved by the Ethics Committee of the Department of Management Sciences, COMSATS University Islamabad Lahore Campus, Pakistan. The patients/participants provided their written informed consent to participate in this study.

## Author Contributions

MAN, MQ, MSN, and IA contributed to writing the original draft of the manuscript, making revisions and reformulation of main theme of the manuscript. KS and AT helped in data collection at first and second stage. All authors contributed to the article and approved the submitted version.

## Conflict of Interest

The authors declare that the research was conducted in the absence of any commercial or financial relationships that could be construed as a potential conflict of interest.
